# Implications of Intravenous and Inhaled Amikacin Breakpoint Reporting for *Mycobacterium avium* Complex Pulmonary Isolates

**DOI:** 10.3390/pathogens14060583

**Published:** 2025-06-12

**Authors:** Christian M. Gill, Robin Chamberland, Getahun Abate

**Affiliations:** 1 Department of Pharmacy, SSM Health Saint Louis University Hospital, Saint Louis, MO 63104, USA; 2 Center for Anti-Infective Research and Development, Hartford Hospital, Hartford, CT 06106, USA; 3 Division of Infectious Diseases, Department of Internal Medicine, Saint Louis University, Saint Louis, MO 63104, USA; getahun.abate@health.slu.edu; 4 Department of Pharmacy, Penn Presbyterian Medical Center, Philadelphia, PA 19104, USA; 5 Network Microbiology, SSM Health St. Joseph Hospital, Saint Charles, MO 63301, USA; robin.chamberland@ssmhealth.com; 6 Department of Pathology, Saint Louis University School of Medicine, Saint Louis, MO 63104, USA

**Keywords:** amikacin liposome inhalation suspension, *Mycobacterium avium* complex, susceptibility testing interpretive criteria

## Abstract

The treatment of *Mycobacterium avium* complex (MAC) remains a clinical challenge as multidrug regimens are needed and may be limited by treatment-related toxicity. The Clinical and Laboratory Standards Institute (CLSI) endorses breakpoints for several agents used for MAC infection treatment. Amikacin has distinct breakpoints for intravenous (IV) therapy and inhaled therapy using amikacin liposome inhalation suspension (ALIS) for MAC pulmonary disease. The purpose of the present retrospective cohort study of MAC pulmonary isolates was to assess the number of amikacin non-susceptible isolates by the IV breakpoints that remain susceptible to the inhaled breakpoints. One isolate per patient per year was assessed and susceptibility was described for amikacin IV, amikacin inhaled, clarithromycin, moxifloxacin, and linezolid per the CLSI. Of the 218 isolates, 94% [204/218] tested as susceptible to amikacin per the IV breakpoints compared with 99.5% [217/218] to the inhaled breakpoints. Of the amikacin IV non-susceptible isolates, 93% [13/14] were susceptible by the inhaled breakpoints. For comparison, clarithromycin was the next most active agent followed by moxifloxacin and linezolid with 97% [211/218], 82% [178/218], and 66% [143/218] of isolates testing as susceptible to each, respectively. These data highlight the importance of laboratories to report both the IV and inhaled amikacin interpretive criteria so that clinicians do not disregard potential therapeutic options for the treatment of MAC pulmonary disease.

## 1. Introduction

*Mycobacterium avium* complex pulmonary disease (MAC PD) remains one of the most common manifestations of non-tuberculous mycobacterium (NTM) lung infections in the United States and globally [1,2]. Treatment for MAC PD remains a clinical challenge as combination therapy is warranted to enhance efficacy and reduce the emergence of resistance [1]. However, this must be weighed against the risk for adverse effects from agents typically employed for MAC PD (e.g., amikacin-related nephrotoxicity) [1]. Novel agents for the treatment of MAC PD have been in development with the most recently marketed in the United States being amikacin liposome inhalation suspension (ALIS) [1,3]. ALIS represents an attractive option as it delivers higher antibiotic concentrations to the site of infection and limits systemic exposure compared with intravenous (IV) administration, potentially enhancing efficacy while decreasing toxicity [1,4]. Clinical and microbiologic data supported the CLSI modifying breakpoints for ALIS (S ≤ 64 mg/L) compared with IV amikacin (S ≤ 16 mg/L) for pulmonary isolates of MAC [4,5]. The CLSI also provides interpretive criteria for other agents considered for the treatment of MAC PD including clarithromycin, linezolid, and moxifloxacin. Contemporary guidelines suggest considering AST results when construction combination therapy as combination therapy has been used to optimize antibacterial efficacy [1].

Recently, the susceptibility profile of NTM isolates from the United States has been published, which provides a robust AST profile that can help support current treatment practices [6]. One of the most important findings was the high geographic and species-level variability in the percentage of MAC isolates that test as susceptible to amikacin, which ranged from 64 to 91% [6]. One point we believe requires consideration is that only IV amikacin breakpoints were reported and thus clinicians must consider both susceptibility breakpoints when selecting therapeutic strategies. Since the ALIS breakpoint is higher than the IV amikacin breakpoint, only reporting the IV breakpoint for pulmonary isolates may lead clinicians away from selecting a viable treatment option for patients.

Herein, we assessed pulmonary isolates of MAC submitted to our microbiology laboratory to assess the number of isolates that were non-susceptible to the IV amikacin breakpoint but remained susceptible at the ALIS breakpoint.

## 2. Materials and Methods

The present study was a retrospective cohort study of patients who had at least one positive pulmonary culture for MAC submitted to our system’s Network Microbiology Laboratory. Isolates were included if they were collected between January 2018 and March 2024. The SSM Health, Saint Louis University Hospital Institutional Review Board reviewed and approved this study (Approval Number, 23-09-2767; Approval Date, 20 September 2023). The need for informed consent was waived.

Multiple isolates per patient were eligible for inclusion; however, only the first isolate per patient per year was assessed. MAC identification was performed using MALDI-TOF MS at ARUP Laboratories per their local standards. MAC susceptibility testing was conducted at one of two outside references laboratories (ARUP Laboratories, Salt Lake City, UT, USA; National Jewish Mycobacteriology Laboratory, Denver, CO, USA) per their standard procedures. Minimum inhibitory concentration (MIC) data were abstracted from the medical record. If antimicrobial susceptibility testing (AST) results were available from two different laboratories for the same isolate, the higher (i.e., more conservative) MIC result was assessed. MICs were assessed for amikacin, clarithromycin, moxifloxacin, and linezolid per CLSI standards (amikacin IV breakpoint: susceptible ≤ 16 mg/L, intermediate = 32 mg/L, resistant ≥ 64 mg/L; amikacin ALIS inhaled breakpoint: susceptible ≤ 64 mg/L, resistant ≥ 128 mg/L; clarithromycin: susceptible ≤ 8 mg/L, intermediate = 16 mg/L, resistant ≥ 32 mg/L; moxifloxacin: susceptible ≤ 1 mg/L, intermediate = 2 mg/L, resistant ≥ 4 mg/L; linezolid: susceptible ≤ 8 mg/L, intermediate = 16 mg/L, resistant ≥ 32 mg/L) [5]. MIC data were described as the percentage of isolates susceptible to each agent and the MIC_50/90_.

The primary outcome was the number of isolates that were susceptible to the ALIS breakpoint but non-susceptible (intermediate or resistant) per the IV amikacin breakpoint. Clinical factors were collected to describe the patient population.

## 3. Results

A total of 218 MAC pulmonary isolates were assessed from 178 patients. Patients were predominantly female (66% [118/178]) with a median age of 70 years old (IQR, 61–78). Pulmonary symptoms were documented at the time of culture for 84% [150/178] of patients. Underlying lung diseases were common with the most prominent being bronchiectasis (65% [115/178]), COPD (49% [88/178]), asthma (17% [31/178]), and cystic fibrosis (5% [9/178]). Seventeen percent [30/178] of patients had received immunosuppressive therapy and 13% [23/178] had active malignancy. Of the cohort, 54% [97/178] of patients received treatment after culture while 26% [47/178] did not start therapy and 20% [34/178] had missing data to evaluate if treatment began.

All isolates were categorized as *Mycobacterium avium* complex. Table 1 describes the MIC_50/90_ and percent of isolates susceptible to each test agent.

**Figure 1:**
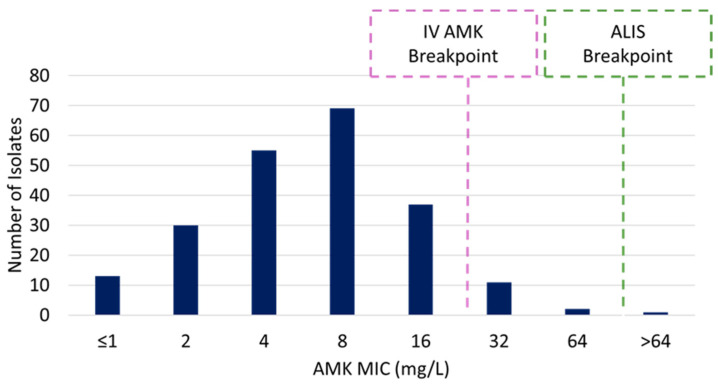
Number of isolates by amikacin (AMK) MIC. IV amikacin breakpoint is susceptible ≤ 16 mg/L; amikacin liposome inhaled suspension (ALIS) breakpoint is susceptible ≤ 64 mg/L.

Of the isolates assessed, 94% [205/218] were susceptible per the IV amikacin breakpoint. Amongst the 6% [14/218] which were non-susceptible per the IV breakpoint, 93% [13/14] remained susceptible per the ALIS breakpoint (MIC range 32–64 mg/L) (Figure 1). For comparison, clarithromycin, moxifloxacin, and linezolid were active against 97% [211/218], 82% [178/218], and 66% [143/218] of isolates, respectively.

## 4. Discussion

MAC PD remains a complex clinical challenge with limited therapeutic options. Novel antimicrobials are needed to provide safe and effective therapy for this challenging infection. As novel therapeutics are introduced, evidence-based breakpoints are needed to guide antimicrobial selection; however, the nuances of these breakpoints must be clearly understood. These data show that despite non-susceptibility to IV amikacin, most isolates remain susceptible to ALIS.

Contemporary guidelines recommend multidrug combination therapy for the treatment of MAC PD to enhance efficacy and reduce the probability of developing resistance to therapy [1]. Therapeutic choices are based on a variety of factors including disease severity and antimicrobial susceptibility testing [1]. Although the CLSI has determined MIC breakpoints for clarithromycin, amikacin, moxifloxacin, and linezolid, clinical outcomes have only been correlated with clarithromycin and amikacin susceptibility testing results [1,5]. MAC PD treatment typically involves clarithromycin, ethambutol, and rifampin, with the addition of IV amikacin in the setting of cavitary disease [1]. Inhaled liposomal amikacin represents an attractive therapeutic option as the direct administration to the site of infection may optimize drug exposure at the site of infection [7]. Similarly, the liposome formulation has been associated with enhanced macrophage uptake, which may also be advantageous due to MAC’s ability to live inside macrophages [8]. Two clinical studies led to the approval of ALIS for refractory MAC where adjunctive ALIS improved the culture conversion rate compared with guideline-directed therapy alone [9,10]. Although disease severity has correlated with outcomes for patients with cavitary diseases [1], approximately 75% of patients in the phase II trial had cavitary disease supporting the use of ALIS in this setting [9]. Although bronchiectasis and/or cavitations on imaging were needed for study inclusion, differences in baseline diagnostics across phase III study sites precluded assessment of outcomes by these disease severity markers [9,10]. The positive clinical outcomes observed in the clinical trials in addition to in vitro susceptibility data led to the distinct clinical breakpoint for ALIS (susceptible ≤ 64 mg/L) compared with IV amikacin (susceptible ≤ 16 mg/L) [4,5].

In the present study, amikacin was highly active in our cohort at both the IV and inhaled breakpoints. A previous analysis evaluated the in vitro potency of a variety of medications against slow-growing NTM including MAC collected from around the US [6]. The proportion of each MAC subspecies that were susceptible to amikacin varied greatly from 64% to 91% [6]. Similar to data from the US, a multicenter study in Italy found 72% and 67% of *M. avium* and *M. intracellulare* were susceptible to amikacin using the same interpretive criteria, respectively [11]. Indeed, species-level identification was not available in our study because it is outside of our clinical laboratory’s standard procedures. Although species-level differences in susceptibility are well described, these isolates were collected as part of routine clinical care. Since contemporary guidelines lack subspecies-level recommendations, this information is not readily determined as our clinical decision making is based on clinical factors and conventional AST [1]. These factors notwithstanding, this represents a limitation of these data and but also suggests our local epidemiology consists of MAC with a higher rate of amikacin susceptibility. Previous data from isolates in the southwestern United States showed similarly high susceptible of MAC isolates to amikacin while isolates from Canada and China showed more variable susceptibility results [5,12,13]. These findings highlight the need for institutions to systematically assess their local epidemiology while considering treatment decisions. Our findings complement those of the aforementioned studies and also raise the clinical concern that both the IV and ALIS breakpoints must be considered when selecting MAC PD therapy. Approximately 20% of isolates from either the US and Italian cohorts were intermediate to amikacin, which would correspond to susceptible per the inhaled breakpoints in the setting of pulmonary diseases [6,11]. If laboratories only report the IV breakpoints, this may lead clinicians away from selecting potentially active therapy, thus highlighting the need for reporting both IV and inhaled amikacin interpretive criteria for pulmonary isolates.

Data from other bacterial infections highlight how susceptibility results portrayed on the AST report highly influence prescribing patterns [14]. Specific to other bacterial infections, the use of selective reporting of antimicrobials on methicillin-susceptible *S. aureus* AST reports led to changes in antimicrobial selection, suggesting what is seen in the reports can lead to changes in prescribing practices [15]. Similarly, the reporting of outdated breakpoints may drive inappropriate use of less effective and more toxic agents in the setting of previous aminoglycoside dosing for Gram-negative infections [16]. These examples from other bacteria highlight the need for laboratories to report the most up to date guidelines to aid clinicians in making optimal therapeutic decisions particularly in complex infections where treatment options are limited. Studies such as these are needed for other areas of infectious diseases in the modern reality of antimicrobial resistance to ensure laboratories are utilizing the most updated breakpoints to inform clinical decisions.

The present study is not without limitations. One consideration in the interpretation of the results is that combination therapy represents the standard of care for MAC PD. Although the exposure response relationships for agents such as amikacin have been evaluated, other agents may offer some activity despite in vitro resistance. Secondly, two laboratories were utilized for MAC AST throughout the study period. Although MIC testing is associated with inherent variability [17,18], only 19 of the 218 isolates had AST conducted at each site, limiting the ability to compare agreement. Thus, analysis of this study used the highest MIC reported, which would be consistent with decision making by clinicians constructing a therapeutic regimen in the clinic.

MAC PD remains a clinical challenge with limited treatment options. In our cohort, amikacin and clarithromycin were highly active in vitro compared with more variable activity observed with moxifloxacin and linezolid. Similar to other published studies, many MAC isolates that test non-susceptible to amikacin per the IV breakpoints remain susceptible to the inhaled ALIS breakpoints. These data highlight the need for laboratories to report both the IV and inhaled amikacin breakpoint interpretations in the setting of MAC PD to avoid clinicians erroneously disregarding a therapeutic option.

## Figures and Tables

**Table 1:** MIC_50/90_ and percent of isolates (*n* = 218) susceptible to each agent.

Characteristic	MIC_50_/MIC_90_	%S
Amikacin IV Breakpoint	8/16 mg/L	94%
Amikacin Inhaled Breakpoint	8/16 mg/L	99.5%
Clarithromycin	0.5/2 mg/L	97%
Moxifloxacin	0.5/2 mg/L	82%
Linezolid	8/32 mg/L	66%

## Data Availability

The original contributions presented in this study are included in the article. Further inquiries can be directed to the corresponding author.

## Abbreviations

The following abbreviations are used in this manuscript:

MAC	*Mycobacterium avium* complex
MAC PD	*Mycobacterium avium* complex pulmonary disease
IV	intravenous
ALIS	amikacin liposome inhalation suspension
CLSI	Clinical and Laboratory Standards Institute
NTM	Nontuberculous mycobacteria
AST	Antimicrobial susceptibility testing
MIC	Minimum inhibitory concentration
COPD	Chronic obstructive pulmonary disease
